# Work-Related Musculoskeletal Disorders among Practicing Plastic Surgeons in India: A Cross-Sectional Survey

**DOI:** 10.1055/s-0045-1802328

**Published:** 2025-01-31

**Authors:** Aditya B. Marathe, Piyush V. Bamnodkar, Ankur S. Karanjkar, Parag B. Sahasrabudhe, Nikhil S. Panse

**Affiliations:** 1 Department of Plastic & Reconstructive Surgery, Byramjee Jeejeebhoy Government Medical College, Pune, Maharashtra, India

**Keywords:** ergonomics, workload, risk, work related musculoskeletal disorders, plastic surgeons in India, surveys in plastic surgery

## Abstract

**Introduction:**

Work-related musculoskeletal disorders (WRMDs) are a less discussed entity in the medical profession, with surgical specialties being more prone to them. Little is known about these types of injuries in plastic surgeons specifically. Data on WRMDs among Indian plastic surgeons are lacking. The goals of this study were to evaluate the prevalence, nature, particular contributory causal factors and behaviors, and potential remedies of these musculoskeletal injuries among plastic surgeons in India.

**Materials and Methods:**

An online voluntary survey was conducted among plastic surgeons in India, collecting their demographics, workload characteristics, musculoskeletal issues, causal factors, and corrective measures, taken using Google Forms. Data were extracted into an MS Excel spreadsheet and analyzed. The prevalence of WRMDs was calculated and the predictors were evaluated with a univariate analysis.

**Results:**

Thirty-three percent of 297 respondents had work-related musculoskeletal injuries or disorders, with the majority experiencing pain (82%), with the neck being the most common site (61%); this was followed by stiffness (61%) and fatigue (52%). The most common causative factors were sustained posture (81%), awkward posture (72%), and inadequate breaks (34%). Age ( *p* = 0.041) and average operative hours per week ( *p* = 0.036) were found to be statistically significant ( *p* < 0.05). The corrective measures cited were stretching exercises, core-strengthening exercises, maintaining proper posture, taking frequent breaks, and yoga.

**Conclusion:**

Plastic surgeons are at high risk of WRMDs, with a significant prevalence in India. Albeit plastic surgeons in India face a higher case load, implementation of ergonomic principles can help in reducing the incidence of these disorders and in preventing the severity of sequelae.

## Introduction

Work-related musculoskeletal disorders (WRMDs) are defined by the Centers for Disease Control and Prevention as “musculoskeletal disorders (injuries or disorders of the muscles, nerves, tendons, joints, cartilage, and spinal discs) in which the work environment and performance of work contribute significantly to the condition; and/or the condition is made worse or persists longer due to work conditions.” Plastic surgeons, especially microsurgeons, have undoubtedly disregarded the warning to adequately recognize the alarming prevalence of inherently preventable WRMDs. WRMDs ensue due to anti-ergonomic postures assumed during complex procedures (such as microsurgery), despite impressive advances across surgical disciplines over decades.

Although surgical working environment has been explored since 1914, the operating room has not altered much over the years, and the scalpel wielder is still subjected to a challenging working environment. 1 It is not unusual for a surgeon to be unaware that a colleague is experiencing surgical work-related musculoskeletal discomfort or injury. The rising frequency of WRMDs among surgeons has been described as “an impending epidemic” 2 and “the tip of an iceberg.” 3

A study of 329 plastic surgeons found that 32% had cervical spine morbidity, 28% required sick leave, and 7% had to change their practice and surgical repertoire. 4 In one survey of 339 surgeons, of which 73% were plastic surgeons, Capone et al found that 77.5% of these plastic surgeons reported symptomatic musculoskeletal injuries. 5 In a study of 865 plastic surgeons, Khansa et al found that 78.3% had work-related musculoskeletal symptoms, 7.3% had to decrease their workload over the long term because of their symptoms, and 9.7% had to take time off. 6

The nature, prevalence, causes, and potential remedies of musculoskeletal problems among plastic surgeons have not received much attention in the literature. There is little research on how work–life balance affects musculoskeletal problems. Our objectives of this study were to assess the prevalence and nature of musculoskeletal injuries in plastic surgeons in India, to identify particular causal factors and behaviors that may contribute to those symptoms, and to suggest potential remedies because this crucial topic has not been thoroughly investigated in India yet.

## Materials and Methods

An online survey to assess WRMDs among plastic surgeons in India was conducted using Google Forms from January 2022 to June 2022 after receiving approval from the institutional ethical committee (ND-Department 0222007–007). The survey was voluntary and anonymous. Only qualified plastic surgeons in India were included in the survey. Data pertaining to demographic and workload characteristics, work-related musculoskeletal injuries, and corrective measures taken were collected with the help of a validated questionnaire ( Supplementary Material , available in the online version). To the best of our knowledge and literature search, there is no established questionnaire to assess WRMDs in plastic surgeons/surgeons. Therefore, a new questionnaire was created and validated. This was then transferred to MS Excel spreadsheet and analyzed.

### Statistical Analysis

Online Google Form data were extracted into an Excel file and the data were cleaned with coding as per the questionnaire. SPSS (Statistical Package for Social Sciences) version 28.0 was used to analyze the data. Incomplete surveys were not included in the data analysis. Frequency and percentage were used to express qualitative data variables. The association between WRMDs and other qualitative data characteristics was determined using the chi-squared test/Fisher's exact test. A *p* -value of less than 0.05 was considered significant.

## Results

A total of 297 responses from plastic surgeons practicing in India were received and analyzed. In all, 37.04% of respondents were up to 40 years of age, followed 31.99% in the age group of 41 to 50 years, 19.87% in the 51- to 60-year age group, 8.75% in the 61- to 70-year age group, and 2.36% were older than 70 years. Male surgeons constituted 81.82% of the survey. Plastic surgeons with a body mass index (BMI) in the range of 25.00 to 29.99 comprised 48.15% of the respondents.

In total, 94.61% of the respondents were right-handed, with 76% using either a no. 7 or 7.5 hand glove. Out of these 297 surgeons, 109 were in private practice/freelancing (36.7%), 98 were hospital employed (33%), 41 were in academic practice (13.8%), 32 had their own hospital (10.8%), and 17 were in group private practice (5.7%).

In terms of the work spectrum and pattern involved, 23.9% were engaged in reconstructive surgery, 2% in cosmetic surgery, and 74% in both reconstructive and cosmetic surgeries. The majority of the plastic surgeons (51.5%) conducted around 100 to 300 surgeries per year, operating for 15 to 30 hours per week (51.18%). Nearly 58.9% volunteers performed regular exercises or physical activity for three to four times a week.

**Fig. 1:**
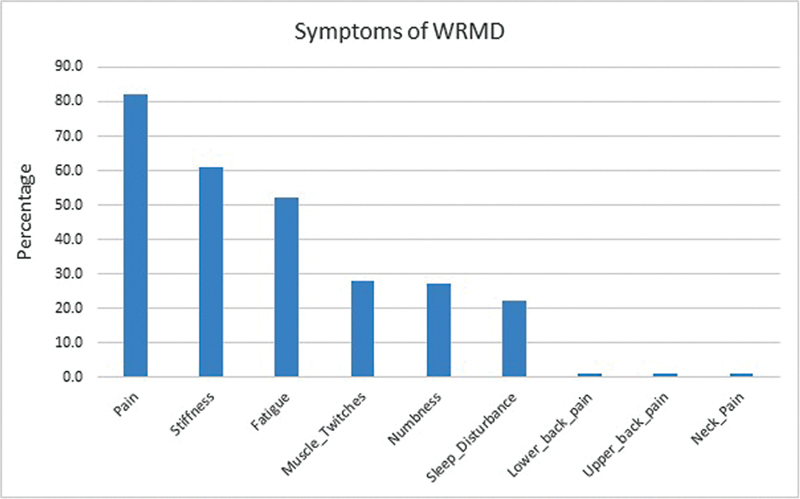
Spectrum of work-related musculoskeletal disorder (WRMD) symptoms experienced by plastic surgeons.

Work-related musculoskeletal injury or disorder *was reported to be present in 100 out of the 297 plastic surgeons surveyed* . In response to the questions on the individual symptoms related to WRMDs ( Fig. 1 ), 82% complained of generalized pain, 61% complained of stiffness, 52% of fatigue, 28% of muscle twitches, 27% of numbness, 22% of sleep disturbance, and 1% each complained of lower back pain, upper back pain, and neck pain. Forty-two percent of the respondents experienced WRMD symptoms in less than 5 years of their practice, 27% in 5 to 10 years, and 31% in more than 10 years. In terms of the specific procedure that most likely would have been the cause of these symptoms, 50% of the surgeons identified microsurgery, 28% general reconstructive surgery, 9% liposuction, 8% hair restoration surgery, 4% brachial plexus procedures, and only 1% craniofacial surgery.

**Fig. 2:**
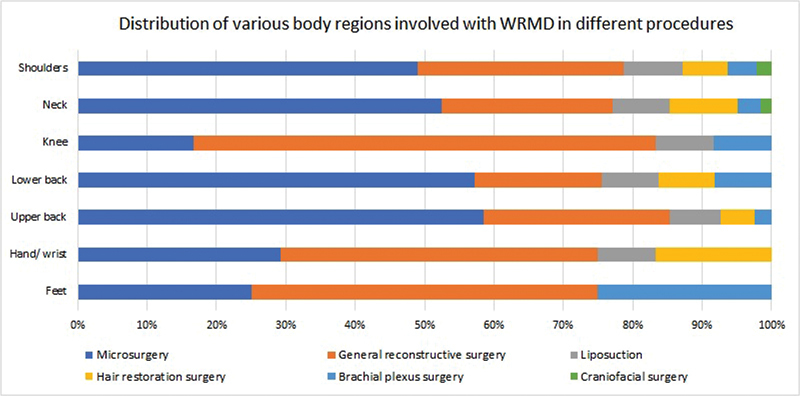
Distribution of various body regions involved with work-related musculoskeletal disorder (WRMD) in different procedures.

**Fig. 3:**
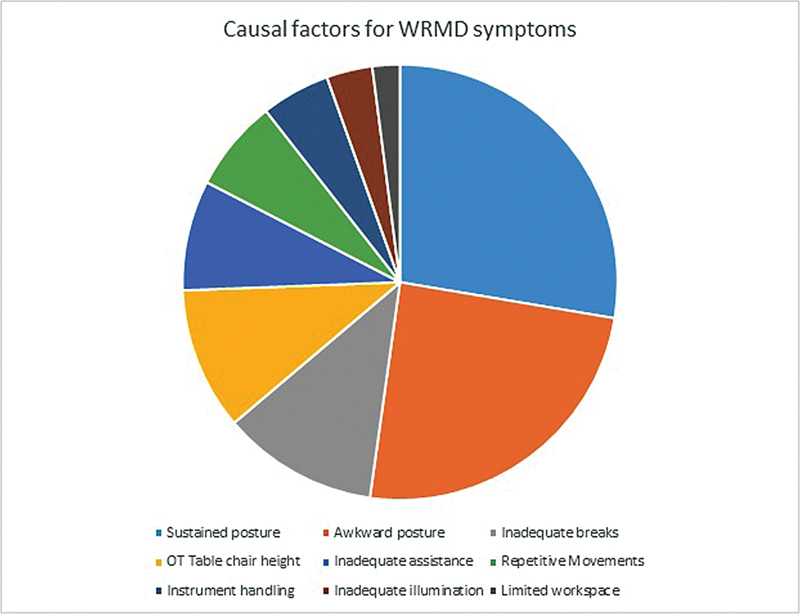
Various causal factors enlisted by plastic surgeons for work-related symptoms. OT, operation theater; WMRD, work-related musculoskeletal disorder.

Sixty-one percent reported the neck to be the most involved site, with the lower back (49%) and shoulder (47%) next in the sequence. The hand/wrist was involved in 24% of the surgeons followed by the knee (12%) and foot (4%; Table 1 ). As per the distribution of the body region involved in WRMDs during various enquired procedures, the neck was most commonly involved during microsurgery (52.5%), followed by general reconstructive surgery (24.6%). The detailed distribution of each region as per associated procedure has been listed in Table 2 and Fig. 2 . The sustained and awkward posture retained by plastic surgeons during the various procedures contributed to WRMDs in 81 and 72% of the respondents, respectively ( Fig. 3 ). Other causal factors reported were inadequate breaks (34%), operating theater (OT) table/chair height (31%), inadequate assistance (24%), repetitive movements (20%), instrument handling (15%), inadequate illumination (10%), and limited workspace (6%). Due to these different types of injuries affecting various sites and resulting from varied reasons, more than 30% of the work was lost in 9% individuals. Twelve percent surgeons experienced 10 to 30% work loss, whereas less than 10% work loss was seen in 20% of them and 59% reported no impact on the magnitude of their work.

**Table 1:** Distribution of WRMD symptoms as per the body region involved in the respondents

Site of injury	Number of cases	Percentage
Feet	4	4.0
Hand/wrist	24	24.0
Upper back	41	41.0
Lower back	49	49.0
Knee	12	12.0
Neck	61	61.0
Shoulders	47	47.0

Abbreviation: WRMD, work-related musculoskeletal disorder.

**Table 2:** Distribution of the body region involved in WRMD during different procedures (%age)

	Microsurgery	General reconstructive surgery	Liposuction	Hair restoration surgery	Brachial plexus surgery	Craniofacial surgery
Feet	25.0	50.0	0.0	0.0	25.0	0.0
Hand/wrist	29.2	45.8	8.3	16.7	0.0	0.0
Upper back	58.5	26.8	7.3	4.9	2.4	0.0
Lower back	57.1	18.4	8.2	8.2	8.2	0.0
Knee	16.7	66.7	8.3	0	8.3	0.0
Neck	52.5	24.6	8.2	9.8	3.3	1.6
Shoulders	48.9	29.8	8.5	6.4	4.3	2.1

Abbreviation: WRMD, work-related musculoskeletal disorder.

Next, the plastic surgeons were enquired about the number of days they had to take leave from their workplace due to either the acute or acute on chronic presentation of the WRMDs. To this, 57% reported of never taking a leave, less than 30 days leave was taken by 37%, and 30 to 60 days of leave by the remaining 6%. Of the 100 surgeons who experienced WRMD symptoms, 72 consulted a health care professional (orthopaedician/physical therapist, etc.) for the management of these injuries. Four individuals underwent surgical procedures for these injuries, including endoscopic lumbar discectomy (1), microdiskectomy (1), vertebral joint replacement (1), and vertebroplasty, angioplasty, renal interventions with dialysis (1). In the end, these surgeons were asked to comment on the different measures they employed in their routine practices to reduce these musculoskeletal injuries.

**Fig. 4:**
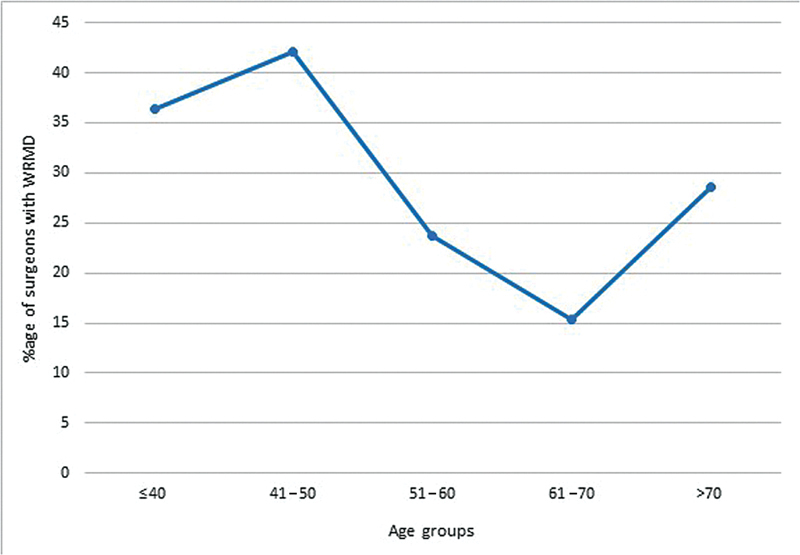
Correlation of work-related musculoskeletal disorder (WRMD) symptoms with age.

**Fig. 5:**
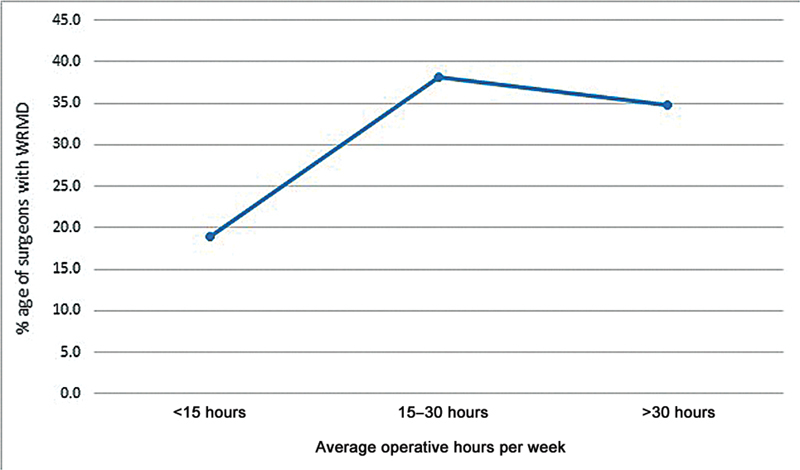
Work-related symptoms strongly associated with operative hours per week.

All the findings from the survey were tabulated in an MS Excel sheet and the data analyzed for univariate analysis of risk factors and *p* -value calculated for the variables in question ( Table 3 ). The age group of the plastic surgeons and their average operative hours per week with a *p* -value of 0.041 and 0.036, respectively, were found to be statistically significant ( *p* < 0.05; Figs. 4 and 5 ). The decrease in trend observed in later age groups was due to a smaller number of plastic surgeons practicing in later age groups. A significant relation was seen between the change in distribution of practice in respondents with WRMDs and different age groups ( *p* = 0.0197 [<0.05]). A significant relation between the plastic surgical procedure and the body regions involved with WRMD symptoms ( *p* = 0.0004 [<0.05]) was observed. The remaining variables analyzed were gender, height, weight, BMI, glove size, hand dominance, practice type, practice distribution, preexisting medical condition, regular exercise performance, and average number of surgeries per year, all with a *p* -value ≥0.05, and hence statistically not significant, that is, not associated strongly with WRMD complaints.

**Table 3:** Univariate analysis of risk factors for musculoskeletal symptoms related to plastic surgery

Factor	Proportion with WRMD	*p* -Value
**Age group**		
≤40 y	36.4	0.041
41–50 y	42.1	
51–60 y	23.7	
61–70 y	15.4	
>70 y	28.6	
**Gender**		
Male	32.5	0.370
Female	38.9	
**Glove size**		
5.5	50.0	0.975
6	28.6	
6.5	33.3	
7	32.8	
7.5	34.6	
8	40.0	
8.5	0.0	
9	0.0	
**Exercise**		
Yes	30.9	0.219
No	37.7	
**Preexistent condition**		
Yes	36.8	0.762
No	33.5	
**Average number of surgeries per year**		
<100	20.0	0.229
100–300	34.0	
300–500	40.5	
>500	26.7	
**Average operative hours per week**		
<15	18.9	0.036
15–30	38.2	
>30	34.8	
**Practice distribution**		
Both	32.7	0.833
Cosmetics	33.3	
Reconstructive	36.6	

Abbreviation: WRMD, work-related musculoskeletal disorder.

## Discussion

WRMDs are one of the unnoticed entities in the medical profession. Surgical specialists, especially those dealing with microscopic and prolonged surgeries, are vulnerable to experience WRMD symptoms. Various studies have been conducted worldwide addressing this important aspect of a surgeon's life, but one encompassing the Indian diaspora was lacking. This cross-sectional survey-based questionnaire was developed to study the varied presentation of WRMDs among practicing plastic surgeons in India. Among the various surgical specialties, plastic surgeons are the most vulnerable to develop this occupational morbidity as described by Soueid et al. 7

In our study, about one-third (33.7%) of the responding plastic surgeons suffered from WRMD symptoms at one point or the other during their career. This trend had a gradual increase with increasing age and a downfall after 60 years. The most common symptoms described were that of generalized pain, fatigue, and stiffness of joints with the neck and lower back involved in most cases. The main causative factors reported in our study were the sustained and awkward posture maintained by the plastic surgeon during the surgery along with higher total operative time per week. This resulted in 70% of the surgeons requiring expert consultation for the treatment of their symptoms, with four of them undergoing various surgeries for the same.

The relatively smaller sample size and comparatively less response rate from the senior plastic surgeons might be the cause of low prevalence rate of WRMDs in our study as compared with other international studies. 5 6 In addition, the decline in the cases at later age group may be due to lesser plastic surgeons practicing at that age.

The small hand size—interpreted from the glove size used by the surgeon during surgery—has been shown to have a strong association with more prevalence of WRMD symptoms in female surgeons and those with small hands. 6 This has also been observed in a study conducted on laparoscopic surgeons by Berguer and Hreljac. 13 The instruments manufactured for operating are of standard dimensions and do not take into consideration the smaller hand sizes of the surgeons resulting in a mismatch, eventually leading to strain on the hand joints due to repetitive use of oversized instruments. 6 In our study, the hand size was not found to be significantly related to the symptoms.

It was interesting to note that, the work-related injuries were not related to the BMI, any preexistent medical or surgical illness, and regular physical exercise. In fact, there were surgeons who had preexisting illnesses and lacked regular physical activity, but still did not experience WRMD symptoms. However, Rambabu and Suneetha have described in their study that physical activity in any form was found to be helpful in reducing MSDs in all age groups. 8 Rață et al in their study on Romanian surgeons have found a higher BMI and sedentary lifestyle to increase the risk of upper back, shoulder, and elbow pain. 9 We found no study correlating preexistent illness and WRMD prevalence.

The neck and lower back were the most commonly involved sites in the survey, similar to those reported in other studies conducted among surgeons of various specialties. Epstein et al in their systematic review on the subject have noted cervical spine disease to be the most common WRMSD. 10 Similar findings were noted by Khansa et al, Capone et al, and by various other authors. 5 6 11 12 Godwin et al conducted an ergonomic assessment of cervical MSD in plastic surgeons concluding that early middle-aged consultants are more prone to cervical morbidity due to prolonged static posture of neck flexion superadded by operating in loupe magnification. 4 The study also mentioned the two significant factors for cervical MSD, the age of the surgeon and hours of loupe usage per week, 4 which are consistent with our findings. Rață et al 9 have described that the surgical workload in the form of “operative hours per week” of the plastic surgeon strongly relates to the shoulder pain and lower back pain. This was in accordance with our study findings that the average operative hours per week were significantly associated with the surgeon experiencing WRMD symptoms. The rest of the symptoms mentioned were similar in distribution to previous studies. 5 6

Ergonomics is the study of matching job requirements and environment to the worker to maximize efficiency, quality, and quantity of work while minimizing WRMDs, fatigue, and overexertion. 14 This concept of ergonomics can also be applied to the operating room and various factors analyzed, influencing the performance of the plastic surgeon while operating. Various authors have studied the operating room as well as surgeon ergonomic perspective, with Patkin describing it for the field of microsurgery, 1 giving a detailed description of visual feedback, hand tremors, and microsurgical skill acquisition. Our survey has not recorded the awareness of ergonomics in practicing plastic surgeons, but there are studies that have discussed ergonomic education and awareness among surgeons of different specialities. 15 16 17

Khansa et al enlisted three common ergonomic errors seen in surgeons: (1) the “head forward” position, (2) sustained and improper shoulder elevation with internal rotation, and (3) pelvic girdle asymmetry. 6 Maximum plastic surgeons in our survey have mentioned a sustained and awkward posture while operating to be a cause of their symptoms. The use of magnifying loupes sometimes with a headlamp and the posture while operating with a microscope predispose the plastic surgeon to have prolonged and static position of neck flexion resulting in strain on the cervical spine. This may be why 50% of our responders with WRMDs attributed their symptoms to microsurgery. The next common error seen is the failure to maintain a proper operating table height, forcing the surgeon to make adjustment in his or her shoulder girdle and leading to upper back pain. In case of prolonged procedures, the surgeon often either tends to shift his or her weight on one of the lower limbs or may need to twist his or her torso to reach the opposite side while operating. These maneuvers result in asymmetry of the pelvis girdle and low back pain. Godwin et al also confirm these findings in their study. 4

The other causal factors enlisted in our survey like inadequate breaks in between prolonged micro-reconstructive procedures, improper assistance, and repetitive movements need special mention, as all of them are preventable and correctable factors that would definitely reduce the surgeon from experiencing MSD symptoms. In a study by Howarth et al, 82.9% microsurgeons changed their positions and 64.1% took frequent breaks to alleviate their symptoms. 11 The U.S. National Research Council has identified repetition, force, and vibration as additional risk factors associated with upper extremity injury. The use of vibratory tools associated with hand surgeries results in the development of thumb arthritis. 5 The vibration and repetitive movements associated with power-assisted liposuction have made the plastic surgeons performing suction lipectomy vulnerable to develop carpal tunnel syndrome. 6

It was seen in the survey that WRMD symptoms did not significantly alter the work volume of the practicing surgeons. Sixty percent of the surgeons never took a leave off work in spite of having symptoms. In the study by Rață et al, the professional activities were affected depending on the part of the body being involved (more for surgeons with neck pain). 9 In many circumstances, surgeons continue to work in spite of pain mainly due to increased workload, professional commitments, and neglect toward self-health. The majority of the plastic surgeons (72%) consulted a professional for their complaints with four undergoing lumbar and cervical spine surgeries. On a similar background, 5% of the cohort of plastic surgeons had to undergo cervical diskectomies in a study on cervical spine conducted by Godwin et al. 4 Khansa et al 6 reported a 4% rate of procedures for the hand for those having arthritis or carpal tunnel symptoms. These outcomes are due to poor ergonomic awareness and poor work–life balance resulting in decreased productivity and inferior operative performance by the plastic surgeon.

Our survey ended with an open-ended question that asked the participants to suggest measures they took to relieve themselves of the MSD symptoms. They elicited stretching exercises, maintaining proper posture, core-strengthening exercises, taking frequent breaks during the surgery, yoga, and many others. These solutions were in full agreement with those reported by Khansa et al. 6 Stretching of the paraspinal muscles frequently causes reduction in neck discomfort. 18 Core-strengthening exercises increase blood flow to the intervertebral disks, result in stabilization of the spine, and improve mood, thereby reducing the perception of pain. 19 Surgical ergonomic education (SEE) is functional at various national and international training centers, but effective implementation and active participation in the learning program by surgeons are the need of the hour. Dedicated ergonomic training as per the specialty needs to be formulated and published.

Recent interventional studies addressing the issue of WRMD have resulted in the development of technologically superior microscopy devices including three-dimensional onscreen microsurgery system (TOMS), Video microscopy, 2D and 3D visualization methods, and the latest being heads-up 3D microscopy (HU3D). Of these, the HU3D display is more comfortable and equally time-efficient without any compromise in image quality and technical feasibility as described by the operators. Although the rest of the methods provide good image quality and comfort, they are not precise and are time-consuming. 20 21 22 23 24 Segal et al have described the use of a biofeedback posture device that helps the surgeon to maintain an upright posture throughout the surgery. 25 Other modifications in the headgear for loupe and lamp, OT tables, and side tables need further development and research to make them surgeon friendly. A summary of previous publications and significant findings are presented in Table 4 .

**Table 4:** Summary of previous publication

Sl. no.	Study	Country	Cohort of WRMD	Respondents	Prevalence (%)	Sites involved	Professional help taken (%)	Intervention required (%)	Preventive/treatment suggestions
1	Capone et al 5	United States	General	Plastic surgeons ( *n* = 236)	77.50	Muscle strain (64%) Neck pain (24.70%)	–	–	–
2	Godwin et al 4	United Kingdom	Cervical Spine	Plastic surgeons ( *n* = 342)	32	Neck	57	18	–
3	Khansa et al 6	United States, Canada, and Norway	General	Plastic surgeons ( *n* = 865)	78.30	Neck pain (66.60%) Shoulder pain (52%)	–	6.7	Core-strengthening exercises, stretching exercises, adjusting operating table height
4	Grant et al 12	Australia	General	Plastic surgeons ( *n* = 11) and other specialties ( *n* = 318)	–	Neck pain (59%) Shoulder pain (55%) [overall]	–	–	–
5	Kokosis et al 26	United States	General	Plastic surgery residents ( *n* = 104)	94.00	Neck pain (54%) Back pain (32%)	–	–	–
6	Rață et al 9	Romania	General	Plastic surgeons ( *n* = 14) Other surgeons ( *n* = 81)	–	Neck pain (71.40%) Back pain (71.40%)	–	–	–
7	Our study	India	General	Plastic surgeons ( *n* = 297)	33.00	Pain (82%) Neck pain (61%) Lower back (49%)	72	4	Core-strengthening exercises, stretching exercises, maintaining proper posture, yoga, etc.

Abbreviation: WRMD, work-related musculoskeletal disorder.

Based on the relevant findings in our study and review of the available literature on the topic, we would like to make certain suggestions to overcome the WRMD symptoms and sequelae:

- Awareness about WRMDs and their sequelae is essential.
- Do not ignore signals from your body and give adequate time to take rest; take frequent breaks between prolonged procedures (30 seconds every 30–40 minutes.)
- Perform regular stretching exercises and core-strengthening exercises to stabilize your spine.
- Be familiar and comfortable with the OT arrangements (i.e., table height, side tables, instrumentation, etc.) and adjust according to your comfort before starting surgery.
- Conscious awareness of one's posture while operating and making necessary change in position help in avoiding a static posture and strain on muscles.
- Having updated knowledge of recent advances, meticulous execution of the steps of surgery, and sound surgical practice is essential for achieving optimal patient outcomes.
- Formal surgical ergonomic training, especially for the trainees at an early age, is essential to prevent further morbidity and develop healthy ergonomic habits.
- Multidisciplinary involvement of ergonomists, occupational therapists, engineers, and policy makers is essential for planning, preparing, and executing a workplace guide.
- Provision for disability-specific insurance should be made in case any untoward event occurs.
- One should be mentally and professionally satisfied with one's work and establish “work–life balance.”

To the best of our knowledge and literature search, this is the first study in India on WRMDs among practicing plastic surgeons. Being a cross-sectional survey, it is an observational study by virtue of its nature and hence has a lower level of evidence. The survey may not be comparative to its international counterparts mainly due to small sample size and confounding factors like preexisting illnesses or addictions not being addressed. In addition, the trainee plastic surgeons, who form a major proportion of the specialty, were not included in the survey. In spite of these limitations, the goals of our study were fulfilled and primary data on the prevalence of WRMDs among plastic surgeons in India were obtained. The experiences of the responders from our study will definitely help us and others for future detailed research into various aspects of this crucial issue. This will aid the future plastic surgeons to develop a healthy and productive career with better patient care, thus fulfilling the mantra of “ *sarve santu nirāmayāḥ* ,” that is, “ *may everyone be free from illness* .”

## Conclusion

With the advent of microsurgery and complex reconstructive challenges faced by the plastic surgeons, WRMDs are now a major concern that needs to be addressed. In this, first of its kind, survey in India for practicing plastic surgeons, we found similar patterns of presentation of WRMDs as compared with their international counterparts along with other behavioral factors suggested by the participants to overcome the problem. Time has now come for the practicing plastic surgeons to look beyond their loupes, toward their operating room, and toward self-care for effective implementation of ergonomics at the workplace, thus providing efficient and prompt patient care along with a long productive career for the surgeon. We plan to conduct a more detailed, analytical study addressing this issue and create awareness among fellow plastic surgeons, especially during their training phase, to reduce the incidence of this preventable disorder.
