# Supplementary material for: Work-Related Musculoskeletal Disorders among Practicing Plastic Surgeons in India: A Cross-Sectional Survey
Source: Indian J Plast Surg. 2025 Jan 31;58(4):276–84. doi: 10.1055/s-0045-1802328 (PMC12396875; doi:10.1055/s-0045-1802328)
Supplement: Supplementary file 1 — Supplementary Material [file 10-1055-s-0045-1802328-s2462916.pdf]

Supplementary Material S1 Questionnaire for work-related musculoskeletal injuries in plastic surgeons in India

1) I am a qualified plastic surgeon practicing in India, and I agree to take part in this survey voluntarily.
   A. Yes
2) What is your age (in years)?
3) What is your gender?
   A. Male
   B. Female
4) What is your height (in cm)?
5) What is your weight (in kg)?
6) What is your glove size?
   A. 5.5
   B. 6
   C. 6.5
   D. 7
   E. 7.5
   F. 8
   G. 8.5
   H. 9
7) What is your hand dominance?
   A. Right
   B. Left
8) Which of the following closely matches your practice type?
   A. Solo private practice/freelancing
   B. Group private practice
   C. Hospital employed
   D. Own hospital
   E. Academic
9) Do you regularly perform exercise/physical activity 3–4 times per week?
   A. Yes
   B. No
10) Which of the following most closely approximates your practice distribution?*
   A. 100% reconstructive
   B. 75% reconstructive, 25% cosmetic
   C. 50% reconstructive, 50% cosmetic
   D. 25% reconstructive, 75% cosmetic
   E. 100% cosmetic
11) Do you have any preexisting systemic/medical illness that you think is likely to have predisposed to musculoskeletal injuries?
   A. Yes
   B. No
12) What is the average number of surgeries you perform every year?
   A. <100
   B. 100–300
   C. 300–500
   D. >500
13) On average, how many hours do you operate per week? *
   A. <15 hours
   B. 15–30 hours
   C. >30 hours

 © 2025. The Author(s).

14) Have you sustained a musculoskeletal injury directly related to your work as a Plastic Surgeon?
A. Yes
B. No

15) What were your symptoms due to the musculoskeletal injuries?
A. Pain
B. Numbness
C. Stiffness
D. Muscle twitches
E. Fatigue
F. Sleep disturbances
G. Other:
H.

16) After how many years of practice did you develop these injuries?
A. <5 years
B. 5–10 years
C. >10 years

17) Which one of the following procedures do you think has most likely contributed to your musculoskeletal injury?
A. Microsurgery
B. Hair restoration surgery
C. Craniofacial surgery
D. Brachial plexus surgery
E. General reconstructive surgery
F. Liposuction
G. General aesthetic surgery

18) Which body part/s were affected due to work related musculoskeletal injuries in your case?
A. Neck
B. Shoulders
C. Upper back
D. Lower back
E. Knee
F. Feet
G. Hand/wrist

19) Which of the following causal factors do you think are most responsible for your musculoskeletal injuries?
A. Sustained posture
B. Awkward posture
C. Repetitive movements
D. Instrument handling
E. OT table height, chair height
F. Inadequate breaks
G. Inadequate assistance
H. Inadequate illumination
I. Limited workspace
J. Other:
K.

20) How much work have you reduced because of these injuries?
A. None
B. <10%
C. 10–30%
D. >30%

21) How many days did you need to take off from work because of this injury (acute or acute on chronic event)?
A. Never
B. <30 days
C. 30–60 days
D. >60 days

 © 2025. The Author(s).

22) Have you ever consulted a professional (e.g., orthopedician/physical therapist, etc.) for management of these injuries.?
   A. Yes
   B. No

23) Did you ever have surgery for this injury?
   A. Yes
   B. No

24) What surgical procedure did you undergo?
25) What measures have you incorporated in your routine so as to get relief from these musculoskeletal injuries?

 © 2025. The Author(s).
